# Patient expectations are a feature of treatment strategy rather than a source of bias

**DOI:** 10.1371/journal.pmen.0000115

**Published:** 2024-09-03

**Authors:** Ralph I. Horwitz, James B. Baker, Mark R. Cullen

**Affiliations:** 1 Aging+ Cardiovascular Discovery Center, Lewis Katz School of Medicine, Temple University, Philadelphia, Pennsylvania, United States of America; 2 Clinica Ai, New York, New York, United States of America; 3 Department of Medicine, Stanford University (Ret), Stanford, California, United States of America; PLOS: Public Library of Science, UNITED KINGDOM OF GREAT BRITAIN AND NORTHERN IRELAND

On June 4, 2024, a group of advisers to the US Food and Drug Administration (FDA) voted that the drug MDMA was not proven effective for treating Post Traumatic Stress Disorder (PTSD). Members of an independent scientific advisory committee voted 9 to 2 that human trials of MDMA did not prove its efficacy. They also voted 10 to 1 that the risks of MDMA, also known as ecstasy, outweigh its benefits.

By way of background, MDMA is a synthetic compound that can evoke euphoria among individuals who take it. The nonprofit group Multidisciplinary Association for Psychedelic Studies (MAPS) has been running MDMA clinical trials using an MDMA treatment protocol that involves a series of psychotherapy sessions along with three sessions in which a team of two therapists administers MDMA. Although the actual mechanism of effect is unknown, it is postulated that MDMA helps patients with PTSD open up to their therapists about traumatic events that they might otherwise be reluctant to confront. Thus, any therapeutic benefit of MDMA for PTSD is embedded in a treatment strategy that includes the drug, the psychotherapy, and the patient’s expectations.

The FDA Advisory Committee reviewed two clinical trials conducted by MAPS in which a total of 194 patients with PTSD received either MDMA plus psychotherapy or a placebo plus psychotherapy [1, 2]. In their application to the FDA, MAPS reported that more than 80% of patients receiving both MDMA and psychotherapy saw significant improvements in symptoms of PTSD, compared to about 60% of patients receiving placebo and psychotherapy. In each trial, twice as many patients receiving MDMA and psychotherapy no longer met criterial for PTSD than patients who received psychotherapy and placebo.

A major concern of the Advisory Committee was the functional unblinding in which patients and therapists could almost always discern whether they had received MDMA or placebo. This was neither unexpected nor hidden by the investigators. In the 2023 trial, the investigators reported that 94% of MDMA subjects correctly guessed they were receiving the psychedelic; and 76% of the placebo patients similarly guessed correctly [2]. The concern about this unblinding was magnified because the outcome was not a “hard” endpoint like death or major morbidity but a “soft” endpoint of a change in a symptom-based rating scale.

It is certainly true that patient expectations have a powerful influence on the outcomes of treatment, and this effect is not limited to psychiatric disorders. It has been 30 years since it was first demonstrated that high adherers to placebo in a randomized controlled trial of beta blockers after heart attack were less likely to die during follow up than poor adherers [3]. Remarkably, the adherence effect in reducing mortality was greater than the effects of the beta blocker itself. These results persisted after adjustment for potential psychological and behavioral confounders.

More recently, Zeng et. al. reported results from a prospective longitudinal study that suggests an association between emotional distress and worse clinical outcomes in patients with advanced non-small-cell lung cancer treated with immune checkpoint inhibitors [4]. The effects were surprisingly large. On the primary endpoint analysis, patients with baseline emotional distress (111 patients) exhibited a significantly shorter median progression-free survival compared with those without distress (116 patients, 7.9 months versus 15.5 months, hazard ratio 1.73, 95% confidence interval 1.23 to 2.43, P  =  0.002). On the secondary endpoint analysis, emotional distress was associated with a lower objective response rate (46.8% versus 62.1%, odds ratio 0.54, P  =  0.022), a reduced 2-year overall survival rate of 46.5% versus 64.9% (hazard ratio for death 1.82, 95% confidence interval 1.12 to 2.97, P  =  0.016) and detriments in quality of life. Further strengthening the findings was the evidence that the emotional distress group showed elevated blood cortisol levels, and that the elevations in this stress hormone were associated with adverse survival outcomes.

As such, it should no longer be surprising when clinical trials confirm what has now been demonstrated repeatedly in the emerging field of Biosocial science: life experience, affect, behavior and expectation influence both the risk for disease and its response to treatment [5, 6]. That is why it is necessary to understand the treatment of PTSD with MDMA not as a drug trial, but as a trial of a treatment strategy in which the effects of psychotherapy, MDMA, and the patient’s expectations, informed by whether the patient received MDMA or placebo, all contribute to their clinical recovery.

A discussion of ways to strengthen the validity of trials that treat diseases such as PTSD and require more than drugs alone is beyond what we can cover in a brief commentary. It is worth noting, however, that it is important to select patient outcomes that are both apposite and reliable. Comparisons with other psychedelic drugs that assist psychotherapy could be considered. A real-world evidence study designed as a target trial with rigorous methods could supplement randomized controlled trial evidence. Ultimately, though, nothing can alter the fundamental reality that patient expectation is a crucial part of the therapy.

There were other concerns about the trials supporting the therapeutic value of MDMA to assist psychotherapy in the management of severe PTSD. It is not clear how durable the effect of treatment is; some patients may have used MDMA after the trial was completed, suggesting that they had persistent symptoms; the total number of treated patients was small; certain adverse cardiovascular events occurred more commonly in MDMA treated patients. We do not presume to judge the decision of the Advisory committee which had access to much more data than we do.

We do wish to challenge, however, the mistaken presumption that patient expectation is a source of bias rather than a feature of the treatment strategy in which patients with severe PTSD are helped to recover from their often debilitating disorder. We understand that a treatment that is dependent on more than the drug alone renders the design and interpretation of randomized trials more challenging. But we cannot allow methodological convention to trump clinical reality. If we deny patients the benefits of treatment because they believe in its benefits, we will also deny them the possibility of a functional life free of the misery caused by their condition. Is that the choice we wish to make?
